# The emerging roles of neutrophil extracellular traps in wound healing

**DOI:** 10.1038/s41419-021-04294-3

**Published:** 2021-10-22

**Authors:** Shuainan Zhu, Ying Yu, Yun Ren, Liying Xu, Huilin Wang, Xiaomin Ling, Lin Jin, Yan Hu, Hao Zhang, Changhong Miao, Kefang Guo

**Affiliations:** Department of Anesthesiology, Zhongshan Hospital, Fudan University, Shanghai, China

**Keywords:** Mechanisms of disease, Experimental models of disease

## Abstract

Delayed wound healing causes problems for many patients both physically and psychologically, contributing to pain, economic burden, loss of function, and even amputation. Although many factors affect the wound healing process, abnormally prolonged or augmented inflammation in the wound site is a common cause of poor wound healing. Excessive neutrophil extracellular trap (NET) formation during this phase may amplify inflammation and hinder wound healing. However, the roles of NETs in wound healing are still unclear. Herein, we briefly introduce NET formation and discuss the possible NET-related mechanisms in wound healing. We conclude with a discussion of current studies, focusing on the roles of NETs in diabetic and normoglycemic wounds and the effectiveness of NET-targeting treatments in wound healing.

## Facts

- NETs may impair wound healing by augmenting inflammation due to pathological conditions.
- Excessive NET formation sustains inflammation amplification and hinders wound healing, probably by affecting wound structures, cellular functions and angiogenesis.
- Anti-NET therapies have exhibited effectiveness in improving wound healing.

## Open Questions

- How are the protective effects of NETs against infection balanced with bystander damage?
- Which types of wounds are not related to excess NET formation? Answering this question may allow us to understand the roles of NETs in wound healing more holistically.
- Are there any other mechanisms by which NETs affect wound healing? Do different types of NET formation vary in their effects on wound healing?

## Introduction

Wound healing is a delicate biological process that includes four overlapping phases (rapid hemostasis, appropriate inflammation, proliferation, and remodeling), and disruption of any phase can result in delayed healing or lack of healing [1]. A plethora of factors affect one or more phases of normal wound healing, including local factors (ischemia, infection, foreign bodies, edema, etc.) and systemic obstacles (diabetes mellitus, hypothyroidism, age, hypothermia, sepsis, medications, obesity, etc.) [2]. At the mechanistic level, prolonged and unbridled inflammation caused by these pathological conditions has been implicated in delayed wound healing [3]. Inflammation occurs soon after tissue damage, and components of the coagulation cascade, inflammatory pathways, and the immune system are subsequently activated to reduce excess blood and fluid loss, clear dead and devitalized tissues, and prevent infection [4]. Circulating neutrophils are among the first cells to be recruited to the wound site; [5] these cells function through phagocytosis, degranulation, and the release of neutrophil extracellular traps (NETs). Recently, NETs have been suggested to be critical for delayed wound healing in several studies [6–9].

In 2004, NETs were described by Brinkmann for the first time in experimental dysentery and spontaneous human appendicitis as structures that bind and kill bacteria [10]. NETs are web-like structures of chromatin filaments coated with histones, proteases, and granular and cytosolic proteins, and the term “NET formation” used to describe the process by which neutrophils produce and release NETs [11].

Some researchers have strongly held that there is little role for NET formation in wounding and repair because it is hard to understand why neutrophils facilitate repair but also release damage-causing NETs [11]. NETs may prevent inadvertent infection, or they may be a result of infection. However, the development of new techniques for imaging living NETs in the wound area and assessment of more accurate markers of NETs have indicated that NETs are critical in impaired wound healing, especially in dysregulated conditions such as diabetes [9, 12]. In this review, we focus on the potential mechanism by which NETs participate in wound healing and the main roles of NETs in diabetic and normoglycemic wounds. We conclude with a discussion of current treatments that target NETs in wound healing and the expectation that novel therapeutic strategies for wound healing will be developed.

## Formation and detection of NETs

**Fig. 1:**
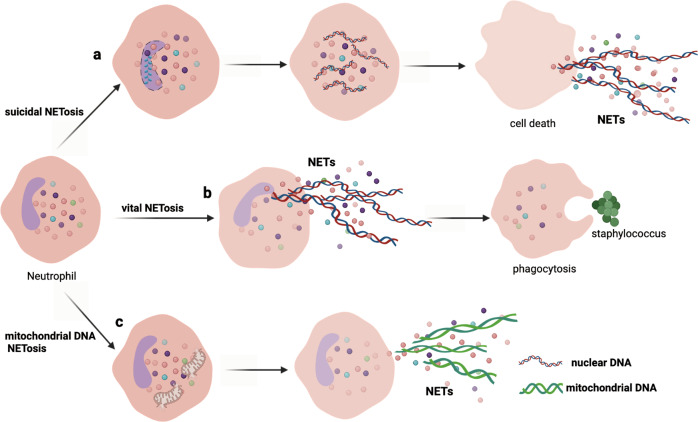
Three types of NET formation. **a** The first type is classic suicidal NET formation, which is characterized by nuclear chromatin decondensation, NET release, membrane rupture, and cell death. **b** The second type is vital NET formation; after releasing NETs, neutrophils are intact and remain phagocytic. **c** The third type is mitochondrial DNA NET formation, which triggers NET formation from mitochondrial DNA but not nuclear DNA.

Upon stimulation, neutrophil elastase (NE) escapes from cytoplasmic granules, enters the nucleus, cleaves histone linker H1, and modifies the histone core, promoting chromatin decondensation [13, 14]. Subsequently, myeloperoxidase (MPO) enters the nucleus to enhance the decondensation of nuclear DNA [15]. Peptidyl arginine deiminase 4 (PAD4) catalyzes histone citrullination, which weakens the binding to DNA for further chromatin decondensation [16]. Later, the nuclear envelope disassembles, and the decondensed nuclear chromatin is released into the cytoplasm of intact cells, mixing with cytoplasmic and granule components. Within 3–8 h after neutrophil activation, NETs are extruded into the extracellular space after membrane rupture and cell death [17, 18] (Fig. 1). NETosis is a classic form of suicidal NET formation and is distinct from the later-discovered vital NET formation, which allows NET release and conventional host defenses to coexist [19].

Yipp and colleagues directly visualized live neutrophils in vivo within minutes during gram-positive skin infections; the cells released NETs during crawling without lysis, which prevented systemic bacterial dissemination [20, 21]. Later, the researchers described this behavior as vital NET formation (Fig. 1). However, vital NET formation might be more closely associated with infection than previously thought because soon after releasing NETs, neutrophils remain alive and can perform other functions in the host response, including chemotaxis, phagocytosis, and killing of bacteria [11].

Notably, viable neutrophils release mitochondrial DNA upon activation in a reactive oxygen species (ROS)-dependent manner [22] that is independent of cell death, which is identified as the third type of NET formation (Fig. 1).

In addition, other types of NETs, such as cloudy NETs, spiky NETs, aggregated NETs (Agg NETs), and bicarbonate-induced Agg NETs, have been described by Daniel and colleagues [23]. Agg NETs can be formed in the context of high neutrophil densities and have a cloudy or clumpy appearance.

Presently, detection of NET formation relies upon several markers, including colocalization of neutrophil-derived proteins and extracellular DNA, citrullinated histones, cell-free DNA, and DNA and neutrophil-derived protein complexes, and on flow cytometric detection of cell-appendant NET components [24, 25]. However, there are still diverging opinions in this field. For example, NETs can be formed in the absence of PAD4 activity and citrullinated histone 3 (H3cit) [26, 27], and the presence of H3cit does not always indicate NET formation; it can also occur in leukotoxic hypercitrullination (LTH), defective mitophagy, and organ injury [28]. We consider opinions regarding these studies with caution. On the one hand, some techniques have been developed to detect NETs, and NET formation can be monitored in real time via intravital microscopy [29] and live cell imaging [30]. Furthermore, immunocytochemical and immunohistochemical analyses of H3cit, MPO, NE, and NET-related proteins have been widely used to illustrate NET formation in many studies. Combinations of multiple detection methods may be more useful for verifying NET formation than single method given that a gold standard marker has not yet been established.

## Mechanism of NETs in wound healing

### NETs affect wound structures

**Fig. 2:**
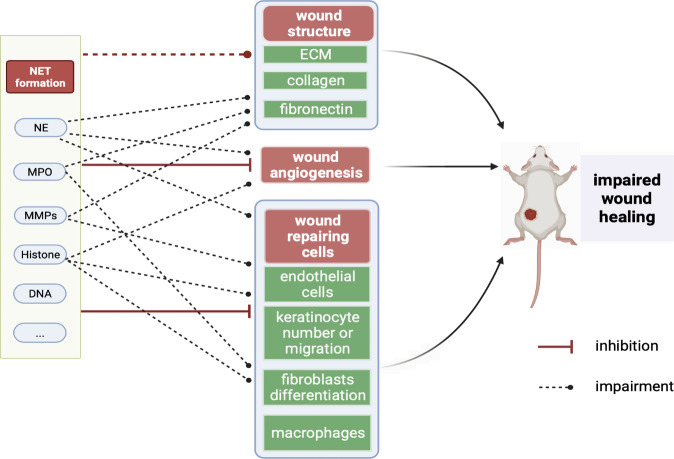
Mechanisms of NET effects on wound healing. Excessive amounts of NET components such as neutrophil elastase, MPO, and MMPs can destroy wound structures, including collagen, fibronectin, and cellular matrix. In addition, NETs impair angiogenesis in the wound area. NETs also affect the number or functions of wound-repairing cells, eventually leading to delayed wound healing.

NETs are structures composed of tangled decondensed DNA, histones, and other granules in the neutrophil cytoplasm. Three different types of neutrophil granules are consecutively generated during neutrophil maturation, including azurophilic granules that contain elastase and MPO, specific granules containing lactoferrin, and gelatinase granules containing matrix metalloproteinases (MMPs) [31]. These granules are released as components of NETs and participate in normal wound repair but harm wound healing when they are overexpressed (Fig. 2).

Excessive NE can degrade some structure- and function-related proteins in wounds, including proteoglycans, collagen, and fibronectin, which disrupts cell connections [32]. Chronic wounds have increased protease levels [33]. Secretory leukocyte protease inhibitor (SLPI) is a serine protease inhibitor that digests NE, maintaining homeostasis. Deleting SLPI increases NE activity and leads to poor wound healing in mice [34]. The application of exogenous SLPI [35] or NE inhibitors [36] can reverse this effect, reduce inflammation, and shrink wounds.

MPO, another NET-associated protein, crosslinks NET proteins and increases NET stability and integrity [37]. MPO has been demonstrated to be a local mediator of tissue damage and the resulting inflammation in various inflammatory diseases that promotes oxidative tissue damage [38]. In crescentic glomerulonephritis, neutrophil-mediated glomerular damage is worsened by excessive extracellular MPO, which may be released during NET formation [39, 40].

Circulating histones are highly cytotoxic and can directly compromise cell membrane integrity and result in tissue damage [41]. Histones integrate into the phospholipid bilayers of cell membranes and change membrane permeability, which may cause an influx of calcium ions and cell death. Histone-mediated complement and Toll-like receptors (TLRs) activation leads to further histone release and inflammation [42]. Histone secretion has even been found to induce more NET formation to exacerbate kidney injury in a recent study [43]. However, histones can be present in the extracellular space not only as components of NETs but also as free histones and as DNA-bound nucleosomes released from dying cells, particularly during necrosis in acute organ injury [44]. Furthermore, how to delineate the effects of free or DNA-bound histones and whether the cytotoxicity varies with different types of histones remain unclear [45].

In the normal wound healing process, MMPs can be digested by nonspecific proteinase inhibitors called tissue inhibitors of matrix metalloproteinase (e.g., TIMP-1) [46]. When MMPs are overproduced during NET formation and cannot be digested to maintain cellular balance, reserved MMPs impede wound healing, and a higher MMP-9/TIMP-1 ratio is associated with poor wound healing [47]. The collagenase MMP-8 breaks down the extracellular matrix (ECM) through a uniquely initiated collagen degradation mechanism [48, 49]. MMP-9 can digest both the ECM and intracellular matrix (ICM), and the latter promotes the removal of DAMP-containing proteins released from damaged cells, which may favor wound healing to some extent [31]. Re-epithelialization commences in the fibrin provisional ECM [50] but may be hindered by excessive ECM digestion by MMPs [3, 46].

To determine whether the NE and other proteins in wound healing are derived from NET formation and not neutrophil degranulation or other mechanisms, Fadini and colleagues washed away unbound proteins and free NET-related proteins with S7 nuclease and quantified DNA-bound elastase and MPO from a NET fraction [12]. These researchers obtained specific and insightful results using this method compared to other routine assessments, revealing that the composition of NETs indeed accounts partly for impaired wound healing.

### NETs affect cellular wound healing

Wound healing is an orchestrated and complicated process involving the spatial and temporal interactions of immune cells and other repair-associated cell types, including endothelial cells (ECs), keratinocytes, fibroblasts and macrophages [51].

ECs are recruited to wounds and participate in angiogenesis, but the effects of NETs on ECs have not been clearly illustrated. NETs act on ECs mainly via directly damage and inhibition of repair proliferation. NET-derived MMP-9 has the capacity to activate endothelial MMP-2, which dysregulates endothelial integrity and function [52]. In addition, circulating histones are highly cytotoxic and directly compromise EC membrane integrity [53], resulting in surrounding tissue damage [54], and NET-accompanied histones induce EC death in a concentration-dependent manner. The administration of histone antibodies decreases NET-mediated cytotoxicity and mitigates epithelial cell and EC death in lung injury [55]. NETs have been reported to evoke the activation and accentuate the thrombogenicity of ECs via IL-1α and cathepsin G, which amplify endothelial dysfunction [56]. Furthermore, endothelial proliferation after tissue damage is of great importance for wound repair, and NETs exert detrimental effects on endothelial migration and tube formation ability [57, 58].

Keratinocytes start migrating to fill a wound defect within a few hours after injury. They migrate through or below the fibrin meshwork and recruit fibroblasts and ECs to form nascent granulation tissue. Keratinocytes proliferate during this process and restore the barrier of the epithelium, which is especially important for larger wounds in which the migration of cells alone is insufficient to close the defect [59]. In a study conducted by Tonello and colleagues, NETs increased keratinocyte proliferation in a concentration-dependent manner through the NF-κB pathway, and low NET concentrations induced faster wound closure with more keratinocytes than the control conditions [60]. In contrast, in the PAD4^-/-^ mouse model, a model with inhibited NET formation, there were no differences in the levels of the proliferation markers Ki67 and TUNEL in keratinocytes in wounds compared to those in wild-type (WT) mice. Thus, these researchers proposed that the migration of keratinocytes was probably inhibited by NETs and resulted in delayed wound healing, but further investigation is needed to verify this conclusion [7]. Recently, a study revealed that the interaction between NETs and keratinocytes enhances *Staphylococcus aureus* skin colonization, which may lead to infections and is known to contribute to lack of wound healing [61]. Thus, how NETs affect keratinocyte wound repair is still elusive.

Fibroblasts provide ECM substances, such as collagen, fibronectin, glycosaminoglycans, proteoglycans, and hyaluronic acid [3, 4]. In the later stage, some fibroblasts differentiate into myofibroblasts, which are contractile cells that help bring the wound edges together. NET components such as chromatin, histones, and MPO induce human fibroblast activation and differentiation into myofibroblasts, moving the tissue toward a fibrotic state [62]. In myocardial infarction patients, NETs have been found to induce the differentiation of monocytes into fibroblasts that accumulate at the wound site and the infarct transition zone, participating in cardiac remodeling [63]. In human skin fibroblasts cocultured with NETs, researchers have also documented upregulated α-SMA mRNA levels and collagen production [64]. However, there is no direct evidence indicating that NETs can activate fibroblasts in wound repair or induce beneficial or poor outcomes.

Macrophages exert different roles at diverse stages of the repair response and orchestrate the natural sequence of wound repair, and conditional deletion of macrophages in any stage results in significantly delayed wound closure [65]. The interaction of NETs and macrophages is incompletely understood but intriguing. On the one hand, macrophages are able to engulf NETs via cytochalasin-D and degrade NETs via cytosolic exonuclease [66, 67]. On the other hand, NETs act on macrophages through various pathways to sustain and exacerbate inflammation. NETs promote macrophage pyroptosis [68], induce a proinflammatory M1-like macrophage phenotype and activate macrophages to synthesize cytokines such as TNF-a and IL-6 [69, 70]. The prolonged presence of M1-like macrophages is not beneficial for the healing process, which is evident in chronic open wounds [71].

### NETs affect wound angiogenesis

New capillaries bring nutrients, immune cells, and oxygen to wounds, supporting wound repair [72]. NETs aggregate in the vasculature and interact with platelets and ECs as scaffolds to form thrombi and promote vaso-occlusion by other means, reducing blood perfusion in wound areas [18, 73]. As a consequence, the clearance of dead tissue is delayed, and ischemia can occur, resulting in impaired wound healing, wound expansion, and superfluous scarring [74]. The host enzymes DNase1 and DNase1L3 independently degrade NETs in serum. In DNase1^-/-^ and DNase1L3^-/-^ mice, scientists have found intravascular clots and entrapped erythrocytes that result in full or partial vascular occlusion in the lungs, liver, and kidneys. The clots are composed of NETs and even in the absence of platelets and pro-coagulation proteins it can be formed independently in the vasculature [75]. DNase therapy resolves NETs and ameliorates local hypercoagulability and clotting-induced hypoxia, contributing to restoration of blood perfusion and acceleration of wound healing. NETs also damage the extant vasculature by other means. Recently, Wang *et al*. reported that overproduction of NETs can activate the cGAS-STING pathway of microglia and induce IFN- and IL-6-induced damage to cerebrovascular integrity [76]. Additionally, NETs affect new capillary formation in the wound site. NE participates in the proteolytic cleavage of some growth factors that are essential for normal wound healing, such as platelet-derived growth factor (PDGF) and the most important positive regulator of angiogenesis, vascular endothelial growth factor (VEGF) [77], thereby impairing wound angiogenesis. Increased NET formation after PAD4 overexpression has been found to lead to poor vascularization and vascular remodeling in a model of stroke, and anti-NET treatments such as DNase1 or PAD inhibition restore angiogenesis [78].

Although debatable, released NETs may also flow through the pathological senescent vasculature to induce reparative vascular regeneration [79]. NETs have been reported to induce angiogenesis in the pulmonary vascular endothelium via ROS-induced TLR4-mediated signaling [80]. Furthermore, NETs can help heal some large wounds by forming plugs to stop bleeding [81]. Collectively, the data indicate that NETs act on angiogenesis in multiple pathways, but the extent of NETs’ function in the whole process of wound angiogenesis is still unclear.

## Roles of NETs in diabetic and normoglycemic wounds

### Diabetic wounds

Multiple factors contribute to poor wound healing in diabetic patients, and wounds become portals for bacterial infection, amplifying cycles of inflammation to hinder wound closure [59]. Thus, wound healing in diabetic patients after surgery warrants further attention. Among various pathological conditions, the role of excess NET formation in wound healing has been studied most often in the context of diabetes.

It has been reported that diabetes predisposes neutrophils to form NETs, which impairs wound healing [7]. Higher levels of H3cit, a biomarker of NET formation, and slower healing rates have found in the wounds of diabetic mice than in those of normoglycemic mice. On the one hand, high glucose concentrations alone prime human and murine neutrophils to produce more NETs in vitro. On the other hand, neutrophils isolated from diabetic patients are more susceptible to form NETs than those isolated from healthy controls and have elevated basal calcium levels that are essential for NET formation. In addition, NET release is strictly dependent on exogeneous glucose and is dependent on glycolysis to some extent [82]. PAD4^-/-^ mice have a reduced ability to form NETs and have been intensively studied in correlation with NET formation. PAD4^-/-^ diabetic mice exhibit faster wound healing than diabetic controls and exhibit low expression of NET-related markers in wound areas. A recent study on the relationship between NET-related markers and insulin resistance in surgical sites after total joint arthroplasty, which is an increasingly common surgery, demonstrated that insulin-resistant subjects had higher PAD4 expression at the surgical site than insulin-sensitive subjects, which may have delayed surgical wound healing [83].

**Fig. 3:**
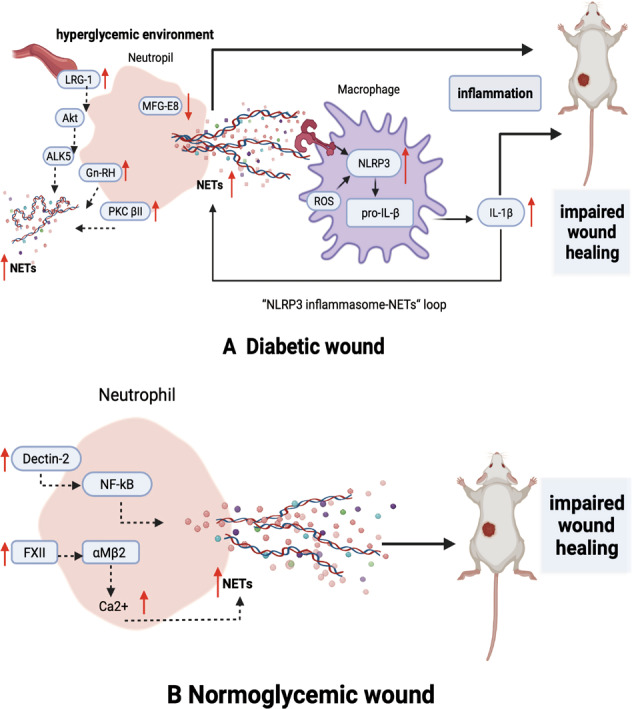
Roles of NETs in diabetic and normoglycemic mouse wounds. **A** In diabetic mouse wounds, NET formation is increased, and inflammation is sustained by the NLRP3 inflammasome-NET loop due to macrophage activation and MFG-E8 deficiency. Increased LRG1 in hyperglycemic mouse blood has been shown to upregulate NET formation via the Akt pathway. PKC βII exhibits hyperactivity in diabetes and contributes to excess NET formation. In addition, GnRH expressed on neutrophils enhances NET formation in the wound area. **B** In normoglycemic wounds, dectin-2 increases the neutrophil inflammatory response and NET formation. Neutrophil-derived FXII modulates neutrophil NET formation by upregulating αMβ2 integrin and increasing intracellular calcium.

The role of diabetes-associated NET overexpression in delayed wound healing and the potential related molecules modulating NET formation have been explored in several studies (Fig. 3A). Excessive NETs produced in diabetic wounds trigger Nod-like receptor protein (NLRP3) inflammasome activation and IL-1β release in macrophages through the TLR-4/TLR-9/NF-κB signaling pathway, sustaining the inflammatory response in situ and impairing wound healing. NETs are the upstream triggers of the NLRP3 inflammasome and activate macrophages, while elimination of NETs benefits wound healing by reducing NLRP3 inflammasome levels and macrophage infiltration [84]. Notably, activation of the NLRP3 inflammasome machinery in macrophages can also promote neutrophils to produce NETs. Researchers have also detected that NLRP3 enhances neutrophil accumulation and NET formation in atherosclerotic plaques [85], indicating that NETs and inflammasomes function in multiple ways.

Milk fat globule epidermal growth factor VIII (MFG-E8) is associated with inflammation resolution, wound neovascularization and wound closure. In a study by Huang and colleagues, MFG-E8 attenuated NET formation and NET-induced NLRP3 activation and IL-8 and IL-18 release to modulate the NLRP3 inflammasome-NET axis [86]. MFG-E8-deficient diabetic mice had abundant neutrophil infiltration, increased NET abundance, poor angiogenesis, and delayed wound closure, suggesting that MFG-E8 can mediate increased NET formation in diabetic wounds. MFG-E8 acts as an endogenous inhibitor of NLRP3, dampens NLRP3 activation and NET formation.

Leucine-rich alpha-2-glycoprotein 1 (LRG-1) is another glycoprotein that regulates neutrophil activation, angiogenesis, epithelial cell proliferation and keratinocyte functions and is critical for timely wound closure. LRG-1 mediates NET formation by activating the Akt pathway through the TGFβ type I receptor ALK5. However, highly elevated LRG-1 concentrations in diabetic patients and mouse serum may skew the aforementioned benefit of LRG-1 in the context of impaired wound healing associated with hyperactive NET formation [87]. Furthermore, LRG-1-deficient diabetic mice are resistant to diabetes-induced poor wound healing, especially during the inflammatory phase, because NET formation is reduced to some extent.

Protein kinase C βII (PKC βII) is a common protein shared by both angiogenesis and the NET formation pathway. Under diabetic conditions, PKC βII hyperactivity induces neutrophils to release more NETs [88]. Upon specific PKC β inhibitor administration, wounds in diabetic mice show reduced NET formation, increased capillary densities and endothelial progenitor cell (EPC) numbers in wounds, and an accelerated healing rate. In addition, the gonadotropin-releasing hormone (GnRH) receptor, which is expressed on the surfaces of neutrophils, participates in diabetic wound healing, and GnRH-enhanced neutrophils undergoing phorbol myristate acetate (PMA)-induced NET formation contribute to wound impairment [89].

Diabetic foot ulcers (DFUs) can be common nonhealing wounds in diabetic patients. In a proteomic analysis of diabetic wound lysates, NET-related proteins, including NE, histone H4, and neutrophil proteinase-3, were enriched in the nonhealing group compared with the rapidly healing group [12]. Furthermore, the concentrations of NET components in the tissue extracts of wound biopsy samples obtained from an independent validation cohort, which divided DFU patients into two groups after a 6-month follow-up according to wound outcome, were higher in the worsening wound group than in the healed or stabilized wound group. Consistent with local wound NET formation, circulating NET-related marker levels were increased in DFU patients. In vivo, diabetic mice exhibited increased NET formation in the wound bed, as determined by multiphoton confocal intravital microscopy, which enables reliable imaging of authentic NET formation. More recently, H3cit was identified as an independent risk factor for wound healing impairment and amputation in DFU patients and was found to correlate positively with the currently applied DUSS and WIfI clinical wound healing scores [90].

Under pathological conditions, the functions of some normal repair factors in the wound microenvironment may be altered, resulting in impaired wound healing due to alteration of the interactions of these factors with neutrophils and NETs. Thus, it is critical that diabetic patients control their glucose levels. Metformin, the first-line glucose-lowering medication for type II diabetes patients, reduces the levels of NET-related components such as elastase and histones in patient serum after 2 months of treatment [91]. Moreover, metformin blunts PMA-induced neutrophil NET formation due to the inhibition of PKC βII translocation from the cytosol to the membrane.

### Normoglycemic wounds

In one study, in normoglycemic WT mice, confocal microscopy substantiated the presence of NETs in excisional wounds, while NETs were absent from unwounded skin. When PAD4 was knocked down in normoglycemic mice, almost no H3Cit was detected in the wounds, which healed faster than those in WT mice. Only 25% of WT controls had all wounds healed on day 14, while the healed wound percentage reached 80% in PAD4^-/-^ mice [7]. In conclusion, NET formation is involved in wound healing in not only diabetic wounds but also wounds caused by aseptic procedures, including surgeries, in normoglycemic patients (Fig. 3B).

Recent studies on nondiabetic mice have offered new insights into the induction of NET formation during wound healing. Dendritic cell-associated C-type lectin-2 (dectin-2), which is expressed on monocytes, recognizes fungi, activates NF-κB, and induces inflammatory cytokine release [92]. It has been verified that dectin-2 affects excisional wound healing by regulating the neutrophil inflammatory response and NET formation [93]. Dectin-2-deficient mice have more collagen deposition, lower levels of MMP-2 and MMP-8, and a shorter healing time than WT mice.

In another study conducted by Stavrou and colleagues, neutrophil-derived coagulation factor XII (FXII) was found to be functionally distinct from hepatic-derived FXII and modulated neutrophils to produce NETs by upregulating αMβ2 integrin, increasing intracellular calcium, and promoting extracellular DNA release [94]. Decreased neutrophil signaling in FXII-deficient mice led to reduced NET formation in the wound and faster healing after sterile full-thickness excision of the dorsal skin.

Taken together, the results of these studies indicate that excess NETs have a negative effect on wound healing, although they are formed by alterations in NET-related upstream modulators under different pathological conditions. Upon exposure to different stimulants, NETs may bind discrepant proteins [27], and not all neutrophil subtypes have the same capacity for NET formation. Low-density granulocytes, which form a subtype of neutrophils in systemic lupus erythematosus, have an enhanced capacity to synthesize NETs and trigger robust endothelial damage [95]. Thus, NETs might be downstream executers in wound impairment to some extent.

## Anti-NET treatments in wound healing

Thus far, several treatments targeting NETs or NET formation have been considered for wound healing (Table 1). The most common treatment is DNase I, which dismantles the scaffold of NET structures, and recombinant human DNase I is cost-effective with no known adverse effects. Upon systemic DNase I administration, diabetic mice show reduced wound areas, enhanced re-epithelialization, and accelerated wound healing [7, 84, 96]. However, DNase I has little effect on histones, elastase, or other components bound to NETs, which may be released into the bloodstream by the simple destruction of the NET scaffold, resulting in proteolytic tissue injuries [29]. Stabilizing NETs and reducing the release of NET degradation products has been found to improve outcomes in murine models of sepsis and to be superior to simple DNase infusion [97]. In contrast, the inhibition of NET production may offer the greatest efficacy in preventing NET degradation product-mediated tissue damage.

**Table 1:** Therapies targeting NETs in wound healing.

Mechanism	Drug/Method	Administration	Wound healing outcome	Ref.
PAD4 inhibitor	PAD4^-/-^ in normoglycemic wound		No H3cit was detected; wounds were healed 80% on day 14 and 25% in WT controls	[7]
	PAD4^-/-^ in diabetic wound		Healed >35% faster, wound area reduced by 28%	[7]
	CI-amidine	10 mg/kg i.v.	H3cit and wound aera decreased	[12]
	Tripeptide (Thr-Asp-F-amidine)	Topically	Wound closure and re-epithelialization accelerated	[98]
DNase 1	Pulmozyme	10 mg/kg i.p.	Superior scar scores and wound closure time	[96]
	Dornase alfa	10 μg i.v. 50 μg i.p.	Wound area reduced by >20% and re-epithelialization by 75%	[7]
	Deoxyribonuclease I from bovine pancreas	Topically	Inflammatory response reduced; re-epithelialization and healing accelerated	[84]
ROS production and MAPK activation	Na_2_S	50 μmol/kg i.p.	NETs reduced; NE activity decreased; wound healing accelerated	[105]
NET formation	GnRH antagonist		Wound size reduced	[89]
	LRG-1 ablation		Wound size reduced	[87]
PKC β inhibitor	Metformin	Orally	NETs reduced	[91]
	Ruboxistaurin	Orally	Wound closure accelerated, nearly complete re-epithelialization by 14 days	[88]
NLRP3 inflammasome-NET axis	Recombinant mouse MFG-E8	500 ng/ml	NETs reduced, NETs-primed NLRP3 inflammasome was inhibited	[86]
NET structure	Clarithromycin	2 μg/ml for 210 min	LL-37 increased, fibroblasts activated, collagen increased	[64]

In PAD4-knockout mice, H3Cit is almost undetectable, and wounds in these mice heal faster than wounds in WT mice [7]. Furthermore, pharmacologically inhibiting PAD4 with CI-amidine rescues wound healing in diabetic mice, providing clinically transferrable evidence that the inhibition of NET formation favors wound healing [12]. Kaur et al. constructed an alginate-gelatin methacrylamide-based scaffold containing Thr-Asp-F-amidine (TDFA), a second-generation irreversible PAD4 enzyme inhibitor, and the topical administration of this substance in a wound area facilitated diabetic wound healing [98]. This is a more feasible method for clinical use than other methods. However, PAD4 is also involved in immune cell function in multiple ways [99], and the impact of PAD4 on infections without enough NET production still needs more exploration. The possibility that PAD4 inhibition may act through mechanisms other than NET formation needs to be considered. For example, PAD4 can be a corepressor for estrogen and thyroid receptors as well as p53 and can modulate cellular differentiation and apoptosis [100]. Additionally, the possibility that NET formation can occur even in the absence of PAD4 activity should be considered [101, 102].

GnRH antagonist treatment also accelerates diabetic wound healing by inhibiting NET formation, and administration of exogenous recombinant MFG-E8, an inhibitor of the NLRP3 inflammasome-NET inflammatory loop, has been demonstrated to ameliorate impaired wound healing in diabetes [89]. Inhibiting the IL-1β pathway by targeting NLRP3 (i.e., MCC950) or inducing IL-1 receptor (IL-1R) blockade with an IL-1R antagonist (anakinra) can also be beneficial to wound healing in diabetes [103]. Additionally, the well-known antioxidant H_2_S has been shown to improve wound healing in diabetes [104]. More recently, intraperitoneal injection of Na_2_S has been found to attenuate NET formation by inhibiting ROS-activated MAPKs and to facilitate diabetic wound healing [105]. LL-37, a key antimicrobial peptide known to promote wound healing [106], is expressed on diabetic NET structures. Clarithromycin administration can enhance LL-37 expression on NET structures and promote wound healing through fibroblast activation and differentiation [64].

## Conclusions and perspectives

Although NETs have bactericidal activity in infections, cells undergoing excessive NET formation exhibit proinflammatory characteristics that contribute to many specific diseases [18, 107]. In this review, we have discussed how the mechanisms by which NETs participate in wound repair, including those involving toxic components of NETs that destroy wound structures, affect healing cells and reduce angiogenesis, sustain inflammation in the wound site and delay wound healing. We have also shown that the existence of NETs in wound sites correlates with poor wound healing in diabetes and even in normoglycemic subjects who undergo sterile surgical operations. Anti-NET therapies, including DNase I, PAD4 inhibitors, H_2_S, and GnRH, have been confirmed to be effective in improving wound healing.

However, there are still some challenges that deserve more attention. First, wound healing is a complicated process affected by multiple factors, and NETs exert mainly negative effects on wound healing according to existing studies. The majority of studies have been based on diabetic wounds, which heal slowly or do not heal in such complicated conditions; few other types of wounds have been examined. Neutrophils produce very few, if any, NETs during the normal healing process [108], and NETs can be formed in the absence of PAD4 [101, 102], which may explain why PAD4 inhibition marginally affects normal wound healing. The role of NETs in normoglycemic wounds has been examined in the context of excess NET formation via the dysregulation of specific pathways. However, the specific roles of NETs in normal wound healing need more research to be fully elucidated.

Second, different triggers of NET formation have been used in the studies conducted thus far, and there are at least 3 types of NET formation, as described in this article. It is difficult to determine whether homogeneous NETs form after stimulation with these factors. Different types of NET formation may facilitate different effects according to different scenarios. Vital NET formation affects mainly infection [11], while the third type of NET formation also has advantages for responses to invading microorganisms and activation of the innate immune system [109, 110]. The majority of studies on wound healing have involved the classic type of NET formation or have not reported the type, while studies on the other forms of NET formation have been limited. It has been reported that Agg NETs containing a plethora of enzymes may serve as inflammatory mediators and degrade proinflammatory cytokines and chemokines, favoring inflammation resolution and wound healing [111, 112]. In addition, Agg NETs sequester NE and protect the ECM from proteolytic attack by NE, as NE can only contact that ECM on the Agg NET surface [113]. Bicarbonate-induced Agg NETs enclose necrotic areas and wounds. Agg NETs play different roles in wound healing than other forms of NETs. However, the previous studies have focused primarily on the situation in which impaired wound healing is associated with increased levels of NET-related proteins and in which excessive NET formation in wound sites impairs the healing process. Research related to the intrinsic mechanisms by which different types of NET formation participate in wound healing is still in its infancy.

In the future, it will be meaningful to take NETs into consideration for wound healing. NETs may be effectors or executors of inflammation resulting from pathological conditions in which they impair wound healing. Manipulating NET formation affects wound healing, and other factors that affect wound healing, such as diabetes, infections, ischemia, and sepsis, are linked with NET formation. Therefore, we believe that NETs may have significant effects in different types of wounds or in complicated wound scenarios. Additional research is needed to describe the balance between the protective effects of NETs in infection and their damaging effects in tissues. It is expected that research on more types of wounds and the intrinsic mechanisms of NETs in wound healing will provide more insights on NET functions and will eventually benefit patients with nonhealing wounds.
